# The value of DWI in predicting the response to synchronous radiochemotherapy for advanced cervical carcinoma: comparison among three mathematical models

**DOI:** 10.1186/s40644-019-0285-6

**Published:** 2020-01-14

**Authors:** Hui Zhang, Yuyang Zhou, Jie Li, Pengjuan Zhang, Zhenzhen Li, Junwu Guo

**Affiliations:** 1 Department of Radiology, The Second Affiliated Hospital of Zhengzhou University, No. 2 Jingba Avenue, Zhengzhou, 450014 Henan Province China; 2 Department of Cardiac Surgery, The Second Affiliated Hospital of Zhengzhou University, Zhengzhou, 450014 Henan Province China

**Keywords:** Cervical carcinoma, Diffusion-weighted MRI, Mathematical model, Radiochemotherapy, Therapeutic response

## Abstract

**Background:**

Diffusion weighted imaging**(**DWI) mode mainly includes intravoxel incoherent motion (IVIM), stretched exponential model (SEM) and Gaussian diffusion model, but it is still unclear which mode is the most valuable in predicting the response to radiochemotherapy for cervical cancer. This study aims to compare the values of three mathematical models in predicting the response to synchronous radiochemotherapy for cervical cancer.

**Methods:**

Eighty-four patients with cervical cancer were enrolled into this study. They underwent DWI examination by using 12 b-values prior to treatment. The imaging parameters were calculated on the basis of IVIM, SEM and Gaussian diffusion models respectively. The imaging parameters derived from three mathematical modes were compared between responders and non-responders groups. The repeatability of each imaging parameter was assessed.

**Results:**

The ADC, D or DDC value was lower in responders than in non-responders groups (*P* = 0.03, 0.02, 0.01). The α value was higher in responders group than in non-responders group (*P* = 0.03). DDC had the largest area under curves (AUC) (=0.948) in predicting the response to treatment. The imaging parameters derived from SEM had better repeatability (CCC for DDC and α were 0.969 and 0.924 respectively) than that derived from other exponential models.

**Conclusion:**

Three exponential modes of DWI are useful for predicting the response to radiochemotherapy for cervical cancer, and SEM may be used as a potential optimal model for predicting treatment effect.

## Background

The synchronous radiochemotherapy has become a standard therapeutic regimen for advanced cervical carcinoma later than IIB [1]. The previous studies [2–4] have demonstrated that DWI is a valuable tool for predicting the early response to radiotherapy for solid tumors, such as rectal, prostate and cervical tumor.

Theoretically, DWI parameters can be acquired on the basis of different mathematical modes, such as IVIM, SEM and Gaussian diffusion models. Das et al. [5] investigated the performance of Gaussian diffusion model (mono- exponential mode) of DWI in predicting the response to treatment in locally advanced cervical cancer, and found that ADC value was a good predictor for pathological response of cervical cancer. Zhu et al. [6] explored the application of IVIM DWI in predicting long-term prognosis in patients with advanced cervical cancer, and found that IVIM parameters were very useful for predicting long-term prognosis. Zhu et al. [7] compared the values of SEM, IVIM and mono-exponential modes of DWI in predicting pathological response to neoadjuvant radiochemotherapy for rectal cancer, and found that α might be more valuable than ADC in predicting treatment effect because it demonstrated better predicting performance and better reliability.

However, up to now, there has been no study to explore which DWI mode is most effective in predicting the response to radiochemotherapy for cervical cancer. Therefore, this study aimed to compare the value of three DWI modes in predicting the response to synchronous radiochemotherapy for advanced cervical cancer, and to determine which mode is most excellent in predicting treatment effect.

## Methods

In the present study, we aimed to systematically compare the value of Gaussian diffusion model, IVIM and SEM modes in predicting the response to radiochemotherapy for cervical cancer in order to establish the most effective DWI mode in predicting treatment effect.

### Study design and population

This is a prospective clinical observational single-center study. This study was approved by the local ethics committee (No. 2013–0112), and written informed consents were obtained from all the patients.

The patients who were suspected to have advanced cervical cancer were enrolled into this study from July 7, 2013 to July 31, 2017. The inclusion criteria included: (1) All the patients had biopsy-proven cervical cancer, and International Federation of Gynecology and Obstetrics (FIGO) stage was IIB-IVA; (2) The mean age was over 18 years old; (3) The patients were scheduled to receive radiochemotherapy, and there was no history of chemotherapy or radiotherapy before the first MR examination; (4) The patients could complete follow-up MRI examinations on time.

Exclusion criteria included: (1) The patients didn’t complete the full course of radiochemotherapy; (2) The patients didn’t complete all the follow-up MR examinations on schedule; (3) There were obvious artifacts on DW images. (4) There was a contraindication to MRI examination (such as allergy to contrast media or claustrophobia).

### MRI technique

The MRI examination was performed on a 3.0 T MRI scanner (Discovery MRI 750, GE Company, USA). For all MRI examination, the patient kept supine position and an empty urinary bladder.

The conventional imaging sequences included: (1) Axial FSE T1WI: TR/TE: 400.0/8.4 ms, slice thickness: 5.0 mm, inter-slice gap: 1.0 mm, matrix size: 320 × 256, number of excitation (NEX) = 2, field of view(FOV): 40 cm × 40 cm. (2) Axial FSE T2WI: TR/TE: 6200.0/136.0 ms, slice thickness: 5.0 mm, inter-slice gap: 0.5 mm, matrix size: 320 × 256, NEX = 2, FOV: 40 cm × 40 cm. (3) Sagittal FSE T2WI: TR/TE: 5000/1000 ms, slice thickness:3 mm, inter-slice gap: 0.3 mm, matrix size: 256 × 256, NEX = 2, FOV: 36 cm × 36 cm.

Multiple b-values DWI was obtained by using single-shot spin-echo echo-planar imaging (EPI) sequence. The main parameters were as follows: 12 b-values: 0, 10, 25, 50, 75, 100, 150, 200, 400, 800, 1000 and 1500s/mm^2^, NEX: 1, 3, 3, 3, 3, 2, 2, 2, 2, 3, 5 and 6, TR/TE: 2000.0/87.0 ms; slice thickness: 5.0 mm, inter-slice gap: 0.5 mm, matrix size: 128 × 128. FOV: 38 cm × 38 cm. The imaging duration was 7 min and 54 s.

The dynamic contrast-enhanced (DCE) images were acquired by using liver acquisition with volume acceleration sequence (LAVA-Flex). The main imaging parameters were as follows: TR/ TE: 4.6/2.6 ms, slice thickness: 3 mm (sagittal imaging) or 5 mm (axial imaging), slice gap: 0.5 mm, flip angle: 12^0^, acceleration factor: 2, NEX: 4, FOV: 36 cm × 36 cm, matrix size: 256 × 256. The contrast media were administered and followed by 20 ml saline flush by using a power injector. The imaging duration for each plane was 37 s.

### Imaging analysis

All the imaging analyses were performed by two radiologists who had 8 and 10 years of experience on pelvic MRI respectively. The two radiologists were blinded to each other’s results. ROIs were manually drawn on DW images at the b value of 800 s/mm^2^ under the guidance of T2WI and DCE MRI. ROI encompassed as much of tumor area as possible, and avoided the recognizable necrotic or cystic areas. The mean value of each imaging parameter for each ROI was calculated. The final value of each imaging parameter was acquired from VOI (volume of interest) for the total tumor. The calculating equation was ƩAV/ƩA, where A was defined as the area for each ROI at each slice of the tumor, and V as the mean value of the imaging parameter for each ROI.

All functional maps of different parameters were calculated by using MADC software on an AW 4.6 workstation provided by the manufacturer (GE Healthcare Company). The data were fitted by using a linear fitting method for Gaussian model, while the data were fitted by using a non-linear least-squares approach for the non-Gaussian models.

The standard ADC was calculated according to the conventional mono-exponential diffusion model by using multiple b-values. The equation was S(b)/S0 = exp.(−b˙ADC) [8].ADC represented the distribution of diffusion-driven displacements.

The equation for intravoxel incoherent motion (IVIM) model was Sb = (1-f) × exp. (−b**·**D) + f × exp. [−b (D* + D)] [8].

Where D was defined as slow component of diffusion, D* as incoherent micro-circulation, f as the volume fraction of the protons linked to the intra-vascular component, and Sb as the signal intensity in the pixel with b value.

The calculating equation for stretched exponential model (SEM) was S(b)/S0 = exp.(−(b˙DDC)^α^) [9].

Where DDC represented the mean intravoxel diffusion rate, and α was related to the intravoxel water molecular diffusion heterogeneity. The range of α was 0~1. When α = 1, the distribution of water molecule diffusion obeyed Gaussian law, while the distribution would no longer obey Gaussian law with increased heterogeneity of tumor.

### Synchronous radiochemotherapy

All the patients received radiotherapy combined with chemotherapy. Radiotherapy regimen included external beam radiation therapy (EBRT) and intracavitary brachytherapy (ICBT). EBRT was administered for 5 weeks at the dose of 1.8 Gy daily (5 days/per week). The total cumulative dose was 45Gy. From the last week of EBRT, ICBT was delivered twice a week with a fraction dose of 6 Gy to point A, and the total dose was 30–40 Gy. The chemotherapy (Cisplatin 40 mg/m^2^/w × 6w) was given for all the patients. The median time interval between first MRI examination and start of radiotherapy was 5 days (range: 3~7 days).

### The classification of treatment effect

Follow-up MRI examination began 4~6 weeks after the start of radiochemotherapy, and subsequently once every 3 months. The follow-up time lasted 1.5 years. Tumor size was determined according to the largest diameter of the lesion measured with electronic calipers on the image that showed the largest axial section of the mass. Two radiologists measured the tumor size independently and were blinded to clinical information. When there was a significant disagreement on the measurement of tumor size between two radiologists, the third radiologist took part in measurement and finally reached a consensus after consultation. When the patient appeared as complete response (CR) at any time point during 12 months, the response assessment ended, and for other patients, response was assessed at 12 months. For the patients who progressed before or those who couldn’t complete the treatment, they were excluded from this study.

The classification of response to radio-chemotherapy included: (1)CR, the tumor disappeared completely; (2) partial response (PR), the tumor had the decrease in diameter more than 30% 12 months after treatment; (3) progressive disease (PD), there was an increase of at least 20% in diameter; (4) stable disease (SD), the tumor appeared as neither sufficient shrinkage to qualify for partial response nor sufficient increase to qualify for progressive disease [10, 11]. CR or PR was classified as the responders group, and SD or PD as the non-responders group.

### Statistical analysis

Statistical analyses were carried out by using SPSS 20.0 package (SPSS Inc., Chicago, IL, USA). We investigated the interobserver agreement on the measurement of imaging parameters by using the concordance correlation coefficient (CCC) according to the following score criteria: good agreement, > 0.75; moderate agreement, > 0.40~0.75; and poor agreement, < 0.40 [12]. We compared the clinical and pathological characteristics between responders and non-responders groups by using *x*^2^ test or Fisher’s exact test. The imaging parameters were compared between responders and non-responders groups by using the unpaired two-tailed *t* test. *P* < 0.05 was considered significant.

A receiver operating characteristic (ROC) was analyzed in order to investigate the capability of imaging parameters in predicting the response to radiochemotherapy. The sensitivity and specificity were calculated on the basis of the cut-off point of each imaging parameter.

## Results

### Patient characteristics

Eventually, 84 patients were included into this study. The clinical and histopathological characteristics for all the patients were summarized in Table 1. The tumor in responders group was smaller than that in non-responders group (*P* = 0.04). There was a higher percentage of poorly differentiated tumor in responders than in non-responders group (*P* = 0.02).

**Table 1:** Patient Characteristics

Parameters	Responders (*n* = 58)	Non-responders (*n* = 26)	*P*-value
Median age (y)^α^	40 (25~67)	38 (29~68)	0.14
Tumor size(no.)			0.04
≤ 4 cm	30 (75.0%)	6 (23.1%)	
> 4 cm	10 (25.0%)	20 (76.9%)	
FIGO stage (no.)			0.06
IIB	8 (13.8%)	6 (23.1%)	
III	26 (44.8%)	10 (38.5%)	
IV	24 (41.4%)	10 (38.5%)	
Pathologic types (no.)			0.10
Squamous carcinoma	48 (82.8%)	21 (80.8%)	
Other pathologic types	10 (17.2%)	5 (19.2%)	
Histologic grade (no.)			0.02
Well//moderately differentiated	18 (31.0%)	20 (76.9%)	
Poorly differentiated	40 (69.0%)	6 (23.1%)	

Note.-^α^The data are expressed as median ages, and the data in the parenthesis are age ranges. The total number within the parenthesis of the column of FGO stage is not equal to 100% because of round off

### Model fitting test

We fitted the different models with the average DWI signals of the tumor respectively. The actual diffusion attenuated signals deviated from the mono-exponential attenuation substantially, which reflected non-Gaussian diffusion behavior. The R^2^ value was 0.954 (*P* = 0.007), 0.926(*P* = 0.008) and 0.712(*P* = 0.006) in SEM model, mono-exponential model and IVIM model.

### The difference in imaging parameters between responders and non-responders groups

The CCCs for standard ADC, D, D*, f, α, DDC were 0.912, 0.891, 0.823, 0.858, 0.969 and 0.924 respectively. The results indicated that there was a good agreement on the measurement of each imaging parameter between two observers.

The imaging parameters derived from three DWI modes were summarized in Table 2. The ADC, D or DDC value was lower in responders group than in non-responders group (*P* = 0.03, 0.02, 0.01), and the α value was higher in responders group than in non-responders group (*P* = 0.03).

**Table 2:** Comparisons of parameters between responders and non-responders group

Parameters	Responders	Non-responders	*P*-value
ADC (10^−3^ mm^2^/s)	0.610 ± 0.072	0.980 ± 0.089	0.03
D (10^− 3^ mm^2^/s)	0.443 ± 0.167	0.843 ± 0.235	0.02
D* (10^−3^ mm^2^/s)	6.891 ± 5.588	8.820 ± 6.456	0.10
f(%)	25.318 ± 9.069	32.356 ± 10.723	0.09
α (unit-less)	0.912 ± 0.043	0.612 ± 0.235	0.03
DDC (10^−3^ mm^2^/s)	0.831 ± 0.141	1.257 ± 0.167	0.01

Note. -The data are expressed as mean value±standard deviation

### The value of the parameters in predicting the response to radiochemotherapy

**Fig. 1:**
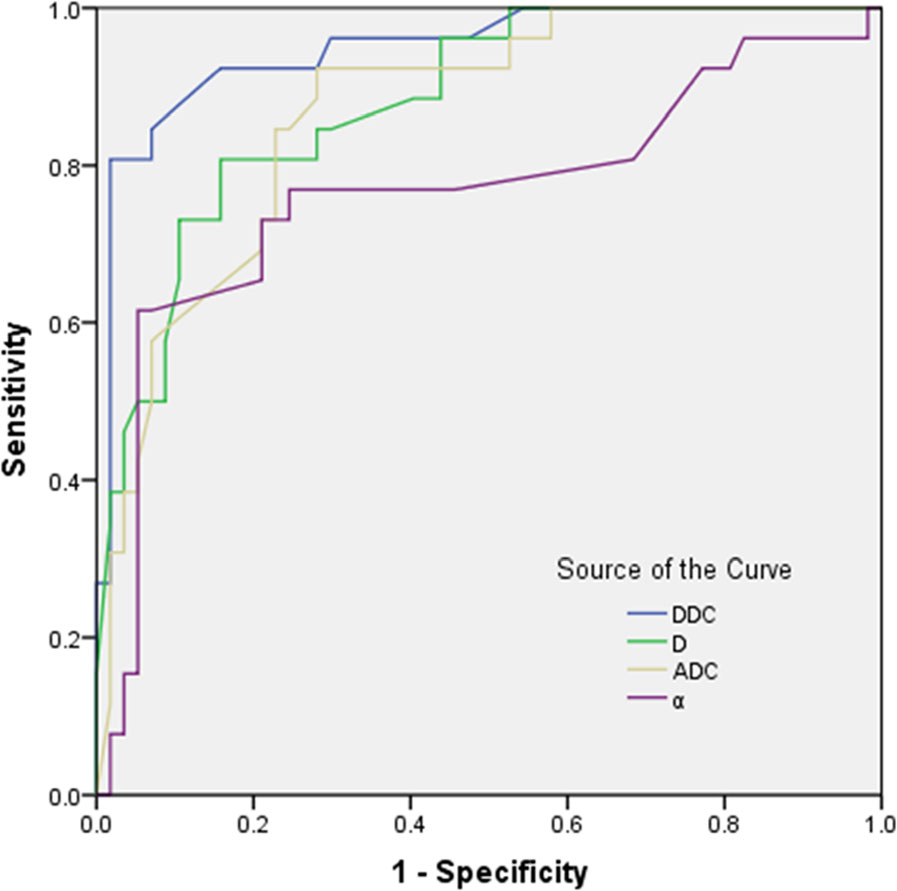
ROCs of imaging parameters in predicting the response to radiochemotherapy. The DDC had the largest AUC (=0.948) compared with ADC, D and α (=0.865, 0.881, 0.766). The cut-off value of DDC was 1.141 × 10^− 3^ mm^2^/s

**Fig. 2:**
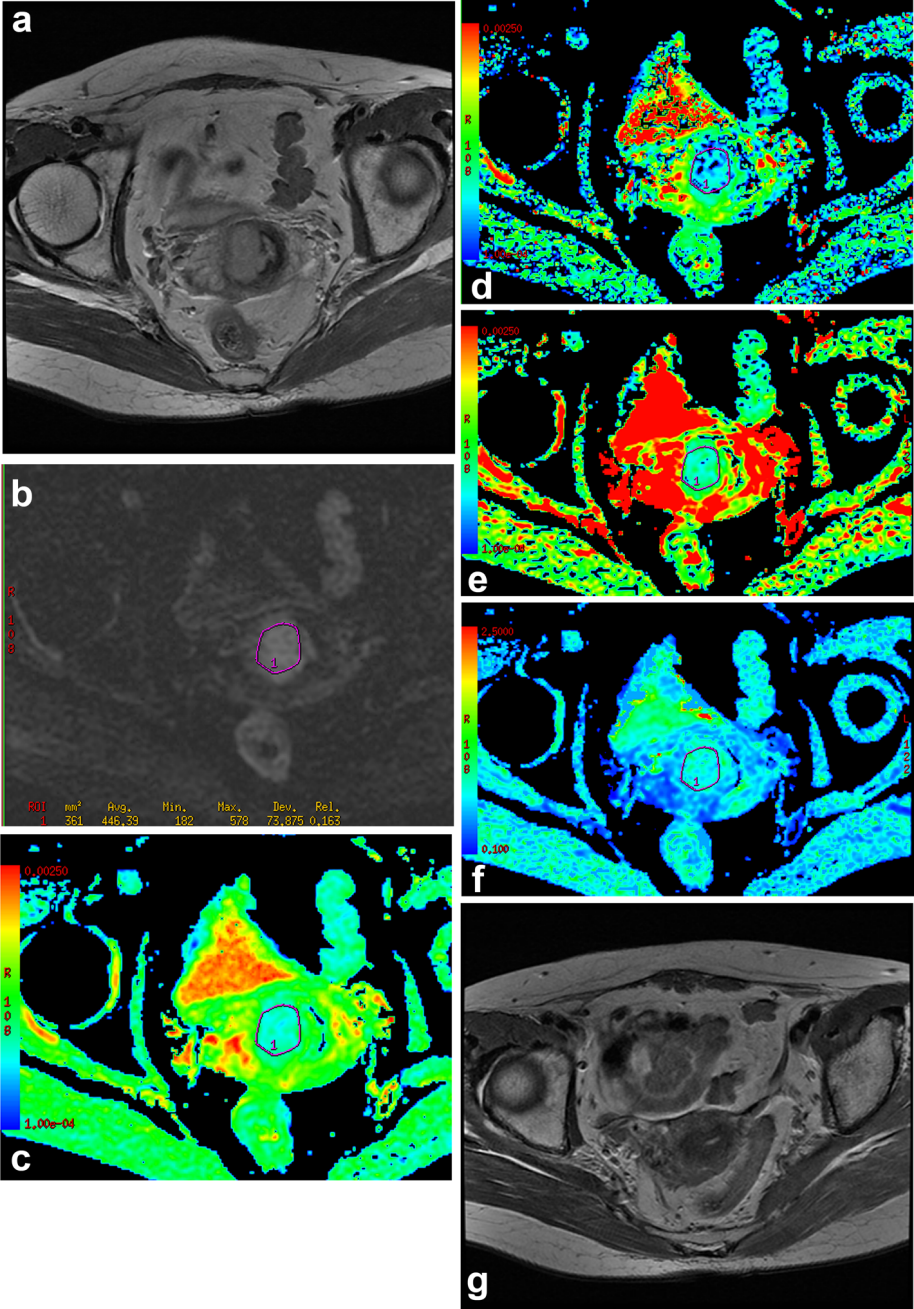
The mean value of DDC in responders group. **a** Axial T2WI, there was an irregular mass in cervix. **b** DWI (b = 800 s/mm^2^), the image showed the location of ROI during the tumor area. **c** ADC map, the final ADC value was low (=0.619 × 10^− 3^ mm^2^/s). **d** D map, the final D value was low (=0.608 × 10^− 3^ mm^2^/s). **e** DDC map, the final DDC value was low (=0.972 × 10^− 3^ mm^2^/s), which was lower than the cut-off value of DDC (=1.141 × 10^− 3^ mm^2^/s). **f** α map, the final α value was high (=0.910). **g** Seven months after radiochemotherapy, the lesion disappeared completely; the patient was classified as responders group

**Fig. 3:**
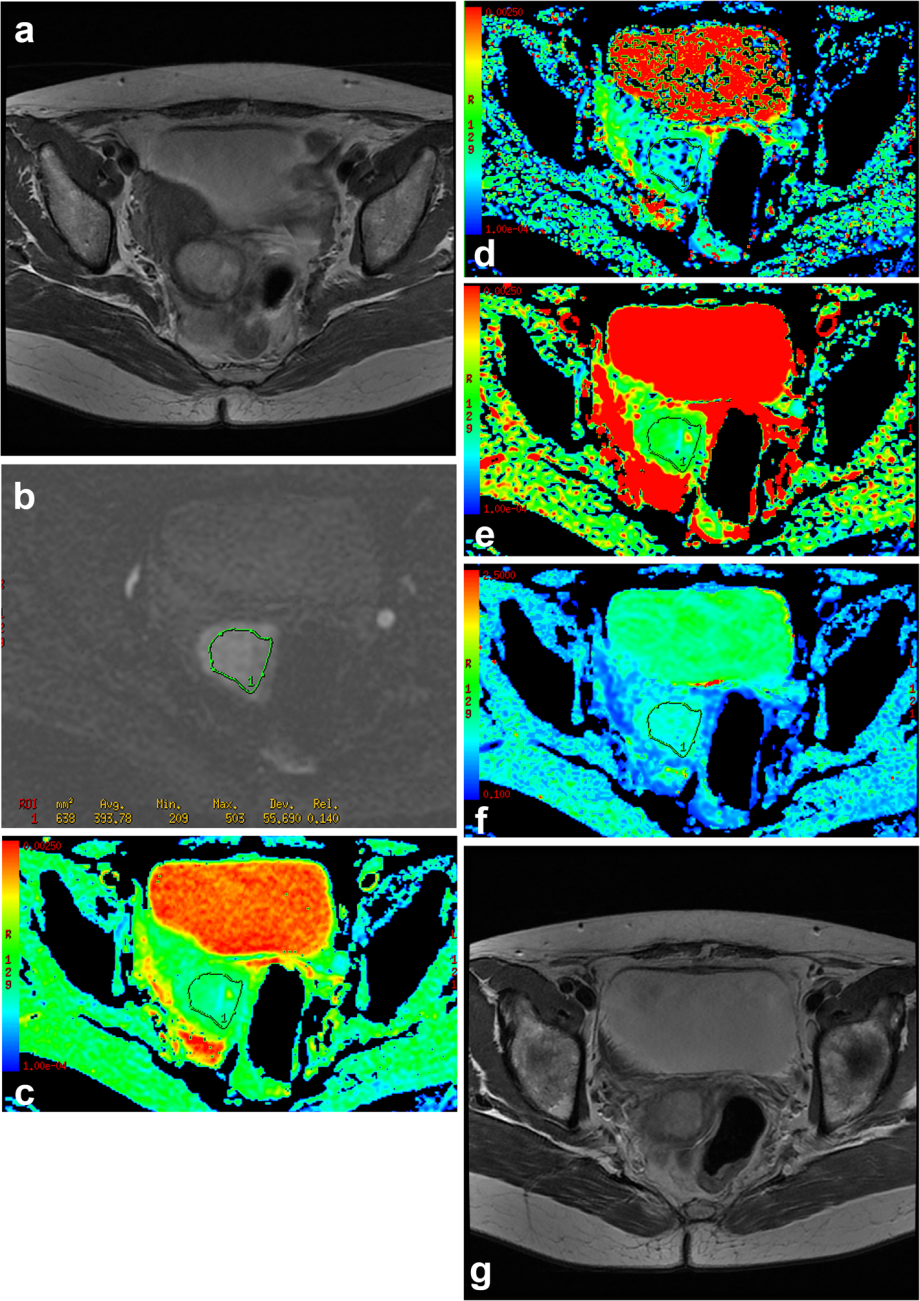
The mean value of DDC in non-responders group. **a** Axial T2WI, there was an irregular mass in cervix. **b** DWI (b = 800 s/mm^2^), the image showed the location of ROI during the tumor area. **c** ADC map, the final ADC value was high (=0.993 × 10^− 3^ mm^2^/s). **d** D map, the final D value was high (=0.876 × 10^− 3^ mm^2^/s). **e** DDC map, the final DDC value was high (=1.237 × 10^− 3^ mm^2^/s), which was higher than the cut-off value of DDC (=1.141 × 10^− 3^ mm^2^/s). **f** α map, the final α value was low (=0.621). **g** Twelve months after radiochemotherapy, the lesion decreased slightly. But the tumor size didnʼt achieve a sufficient shrinkage to qualify for partial response, therefore, the patient was classified as non-responders group

The areas under the curves (AUCs) of ADC, D, α and DDC in predicting the response to radiochemotherapy were 0.865, 0.881, 0.766 and 0.948 respectively (Table 3). DDC had a larger AUC compared to ADC (*P* = 0.02), D (*P* = 0.03) or α (*P* = 0.04) (Fig. 1). The cut-off point of DDC was 1.141 × 10^− 3^ mm^2^/s, which yielded 93.3% sensitivity and 86.5% specificity (Figs. 2 and 3).

**Table 3:** The diagnostic performances of parameters in predicting responders

Parameters	Cut-off value	AUC(95%CI)	Sensitivity	Specificity
ADC (10^− 3^ mm^2^/s)	0.752	0.865 (0.753–0.902)	85.1%	67.4%
D (10^−3^ mm^2^/s)	0.532	0.881 (0.783–0.923)	82.1%	73.3%
α (unit-less)	0.734	0.766 (0.654–0.834)	76.3%	78.4%
DDC (10^−3^ mm^2^/s)	1.141	0.948 (0.812–0.976)	93.3%	86.5%

Note.- *AUC* The area under ROC curve, *CI* Confidence level

## Discussion

DWI is very helpful for predicting the response to therapy or viability of therapy. Razek et al. [12] found that ADC map was an excellent method for differentiation between the viable and necrotic parts of head and neck tumors, and they also found that diffusion tensor imaging (DTI) was very valuable in differentiation between residual tumors and post-radiation changes [13]. In addition, DWI is very useful in differential diagnosis of different tumors. Razek et al. [14] demonstrated that a combination of arterial spin label (ASL) - and DTI-derived metrics of the peritumoral part could be used for differentiation between glioblastomas and solitary brain metastasis.

It is generally known that DWI models include a Gaussian diffusion model and non-Gaussian diffusion models. A Gaussian diffusion model (mono-exponential model) is considered as free diffusion (Brownian diffusion) under the ideal condition and simply described as average diffusion value [15]. However, the water molecular diffusion in tissue deviates from Gaussian diffusion because that the diffusion is usually restricted by cell membranes, fibers or electric charges at the proteins. Therefore, Gaussian diffusion model can’t reflect the real situation of tissue or tumor micro-structure. A non-Gaussian model (such as IVIM or SEM) can reflect the real distribution of water molecular in human tissue more accurately [9, 16]. The study on the relationship between non-Gaussian models and the response to radiochemotherapy is very useful for the design of treatment regimen.

The standard ADC value acquired by us had a higher diagnostic efficacy in predicting the response to radiochemotherapy. For example, Das et al. [5] showed that the AUC of ADC in predicting the response to radiochemotherapy was 0.814, while our study showed that the AUC of standard ADC was 0.865. The possible reason is that ADC value is calculated on the basis of two b values in the study by Das et al., while the standard ADC value is calculated on the basis of 12 b-values in the study by us. The selection of more b values can avoid selection bias and thus achieve more reliable results even for a mono-exponential model [17].

This study showed that there were significant differences in ADC, D, α and DDC values between responders and non-responders groups. Among the imaging parameters, DDC had the highest capability of predicting the response to radiochemotherapy. This result was similar to the study by Bedair et al. [18], who found that there was an inverse correlation between DDC value and therapeutic response. The possible reason is that DDC reflects the mean intravoxel diffusion rate in the tumor. The tumor with higher DDC value frequently has necrosis and poor oxygenation, which results in the resistance to radiochemotherapy [18]. In addition, this study showed that responders group had higher α value than non-responders group. The α value reflects the micro-structural complexity of the tumor, such as cellular pleomorphism, vascular heterogeneity and presence of microscopic necrosis. The lower α value seen in non-responders group indicates a more heterogeneous microenvironment within the tumor area [19, 20].

This study demonstrated that there was no significant difference in D* or f value between responders and non-responders groups. Similarly, Liang et al. [21] investigated the value of imaging parameters derived from IVIM mode in predicting the response to radiotherapy for rectal cancers, and found that there was no significant difference in D* or f value between pathological complete response (pCR) and non-pCR groups. Xiao et al. [22] investigated the value of IVIM parameters in predicting the early response to induction chemotherapy for nasopharyngeal carcinoma, and found that D* and f value showed no significant differences between effective and ineffective groups. These results indicate that perfusion-related parameters may not play an important role in predicting responders after treatment. However, it is noteworthy that D* has some technical shortages, such as data instability and its dependence on signal noise ratio (SNR) [23], which may also be the main reasons why there is no significant relationship between D* value and treatment effect.

This study investigated the reproducibility of the imaging parameters derived from three DWI modes. Among the imaging parameters that had significant differences between responders and non-responders groups, the parameters derived from SEM model had the best repeatability. This result is similar to a previous study by Jerome et al.,who found that SEM outperformed other models [24]. In addition, we found that significantly better fitting of DWI signals could be acquired by non-Gaussian diffusion models, with the exception of IVIM model. Therefore, the imaging parameters derived from SEM may be used as a potential optimal model for predicting treatment effect.

Except for IVIM, SEM and mono-exponential diffusion model, other advanced diffusion imaging models, such as DTI and diffusion kurtosis imaging (DKI), have been used in differentiation between benign and malignant tumors [25, 26]. However, the role of DTI or DKI in predicting the response to radiochemotherapy for cervical cancer is still unclear and needs to be furtherly investigated.

The limitations to this study were as follows: (1) In this study, 12 b-values were selected, which might not be optimal for all the mathematical models, therefore, the further study is necessary to optimize the design of b-values for different exponential modes [27]. For example, SEM was analyzed on the basis of 12 b-values in this study, while the previous studies [28, 29] acquired the similar results by using approximate 4–5 b values. Therefore, the selection of b values might be not optimal for SEM mode. (2) The number of the patients in non-responders group was relatively small, which might bring about subtle selection bias. (3) The f values were not T2 corrected and thus could have been influenced by the T2 relaxation times of blood and tissues [30].

## Conclusions

In conclusion, this study shows that both Gaussian diffusion model and non-Gaussian diffusion models of DWI are helpful for predicting the early response to radiochemotherapy for cervical cancer. The wider use of non-Gaussian diffusion models in assessing treatment effects may provide more important information on the prognosis of cervical cancer. Especially, SEM mode has the highest potency for discriminating responders from non-responders after radiochemotherapy.

## Data Availability

The datasets used and/or analyzed during the current study are available from the corresponding author on reasonable request.

## Abbreviations

ADC: Apparent diffusion coefficient
AUC: Area under the curve
CCC: Concordance correlation coefficient
CR: Complete response
DCE: Dynamic contrast enhanced
DWI: Diffusion-weighted imaging
EBRT: External beam radiation therapy
FOV: Field of view
HE: Hematoxylin and eosin
ICBT: Intracavitary brachytherapy
IVIM: Intravoxel incoherent motion
MRI: Magnetic resonance imaging
NEX: Number of excitation
PD: Progressive disease
PR: Partial response
ROC: Receiver operating characteristic
ROI: Region of interest
SD: Stable disease
SEM: Stretched exponential model
TR/TE: Repetition time/time to echo
VOI: Volume of interest
